# Improvement of water harvesting performance through collector modification in industrial cooling tower

**DOI:** 10.1038/s41598-022-08701-3

**Published:** 2022-03-18

**Authors:** Ji Yeon Kim, Jong Hoon Kang, Jong Woon Moon, Sung Yong Jung

**Affiliations:** 1 Department of Mechanical Engineering, Chosun University, 309 Pilmun-Daero, Dong-gu, Gwangju, 61452 South Korea; 2 POSCO, Technical Research Laboratories, Iron & Steel Process Engineering Research Group, Pohang, South Korea

**Keywords:** Hydrology, Sustainability

## Abstract

Shortages of freshwater have become increasingly common around the world, and various studies have been conducted to solve this problem by collecting and reusing the water in nature or from factories and power plants that produce large fog plumes. Although the shape of a collection screen is strongly related to its harvesting performance, only flat meshes have been considered in previous studies, and research on the effects of collector structure shapes is severely lacking. In this study, we proposed modified collector structures improving harvesting performances in industrial cooling towers. The screen shape was modified in three steps. First, a concave shape was adopted for the mesh screen to increase the aerodynamic characteristics of the collection structure. Next, a sidewall was installed to collect additional fog from defected flows generated by the concave structure. Finally, to reduce loss during the draining of collected water droplets, the discharge direction of the fog flow was changed to follow the same direction as fog-laden flows in nature. Our results are expected to be useful for collector design in terms of increasing harvesting efficiency in various industrial fields in the future.

## Introduction

Freshwater is an essential resource for maintaining human life, raising livestock, irrigation, and urbanization in various industries^1^. Freshwater on the earth’s surface is extremely limited and accounts for only 2.5% of the earth’s total water content. Although water covers 70% of the surface of the earth, approximately 97.5% of this water is salt water. Additionally, glaciers and icecaps in polar and alpine regions account for 68.7% of the freshwater. Therefore, readily available freshwater only accounts for approximately 1% of the total water content on earth^2^. In the twenty-first century, one major environmental disaster is a shortage of freshwater caused by the pollution. Shortages of freshwater cause reductions in economic growth and overall quality of life. Additionally, problems such as famine caused by shortages of drinking water will worsen over time. To raise awareness of this global water crisis, UNESCO launched the International Year for Water Cooperation in 2013^3^ and the UN proclaimed the period from 2005 to 2015 as the International Decade for Action of “Water for Life”^4^.

To overcome freshwater shortages, harvesting water in the air has been proposed as a simple and economical approach to obtaining freshwater. In general, the moisture contained in air can be collected using two main methods. The first is fog harvesting, which directly collects small droplets floating in fog flows. The other is dew harvesting, which collects phase-changed moisture through vapor condensation on surfaces. It is known that both water harvesting methods are strongly influenced by surface wettability and the surface structural features^5,6^.

Recent research has focused on fog harvesting because it is energy free, unlike dew harvesting, which generally requires input energy to reduce surface temperatures. Fog harvesting generally involves the collection of water through a two-step process. The first step is to capture droplets on the surface of a collector, which is affected by the collector shape, droplet size, mesh wire radius, and shade coefficient (*SC*). In this stage, capturing performance is highly affected by wettability, which has been intensively studied for atmoshperic fog harvesting. In the second step, the droplets captured on the surface are transported to a reservoir. This process can be improved under certain conditions when the loss caused by evaporation and re-entrainment into fog-laden flows is minimized^7^. Additional losses in industrial fog harvesting occur as a result of fallback caused by gravity during this step. This additional loss leads to lower performance in industrial fog harvesting compared to the performance in nature. Therefore, for practical applications of fog harvesting in various industries, a new strategy is required to improve the performance of this second step.

Several researchers have demonstrated the practical potential of fog harvesting for freshwater resources in various regions, including the Pacific coast of northern South America, coastal deserts of Peru, Sultanate of Oman, Canary Islands, Morocco, Namib Desert, and hills of Nepal^8–10^. Most studies have installed huge nets using a double-layer Raschel mesh with two poles arranged vertically for fog capture^11–13^. However, a Raschel mesh has disadvantages in that it is difficult to create a double layer with similar *SC*s and to purchase Raschel meshes with the same dimensions in different countries. Additionally, such meshes are easily torn by strong winds. Furthermore, it is difficult to modify the wettability of such meshes. Therefore, many studies have been conducted using metal meshes instead of Raschel meshes. Recently, studies on wettability have been conducted to improve harvesting performance. Park et al. proposed improving fog harvesting capabilities by using commercial metal meshes with surface wettability modification^14^. Surfaces with mixed wettability patterns were developed by mimicking a desert beetle’s back, which consists of a hydrophobic surface covered with hydrophilic bumps, to adapt to areas with annual rainfall of less than 12 mm and enhance water collection^15–17^. These surfaces have demonstrated better harvesting performance than surfaces with uniform wettability^18–21^. Li et al. improved the fog collection efficiency by modifying the wettability of polyester filter nets with hydrophilic and hydrophobic^22^. Sarafpour and Youssefi studied the effect of fabric texture on various non-mesh fabrics through a nickel-phosphorus electroless coating method^23^.

Previous studies on fog harvesting have mainly been conducted in nature, especially in areas with dry fog, such as deserts and other areas with low rainfall and severe temperature differences, as well as in humid areas such as alpine regions. Therefore, most previous studies have limitations in that their harvesting performance varied depending on the climate and geographical location. To overcome this limitation, industrial cooling towers can be used for fog harvesting as alternative water source. White plumes from cooling towers have recently received significant attention from an environmental perspectives^24–26^, because a large amount of the water used to cool hot substances is discarded through cooling towers. Additionally, industrial fog harvesting can reduce resident complaints because the discarded fog appears similar to smoke from a factory. Several studies have been conduted on collecting freshwater from industrial cooling towers using metal meshes with surface wettability modification^27,28^. In our previous study, a metal mesh was used to study the water discarded from cooling towers^29^. Experimental analysis was performed by varying the mesh angle, and the highest fog collection efficiency was obtained at an angle of 30°. Additionally, chemically modifying the surface wettability of a mesh can improve fog collection efficiency.

Although several studies have investigated the collection of freshwater from fog-laden flows in various industries, to the best of our knowledge, research on the effects of collector structure shapes, which are strongly related to harvesting performance, is severely lacking. In this study, we proposed a method to solve the fresh water shortage problem by collecting and reusing water discarded from industrial cooling towers.

First, a concave shape was fabricated with consideration for aerodynamic efficiency and compared to a flat mesh. Second, a side wall was installed on the concave shape to capture deflected flows. Finally, the discharge direction was changed to compensate for the limitations of fog collection in industrial cooling towers. Comparative analysis of the effectiveness of the proposed structure modifications was performed using a downscaled industrial fog harvesting device simulating a cooling tower in a steelmaking factory.

## Materials and methods

### Modification of mesh wettability

In this study, commercial Al meshes (Goodfellow, UK) with wettability modification, which is known to be a robust and simple method, were adopted by considering the material and modified surface durability^30^. Table 1 contains the specifications of the used meshes including the wire diameter, opening distance, porosity, and shade coefficient. These values were tested and provided by the manufacturer. The Al mesh was immersed in a 1 M sodium hydroxide (NaOH) solution for 1 min and then the residual liquid was removed using deionized (DI) water, followed by etching in a 2 M hydrochloric acid solution for 3 min. The mesh was then immersed in the NaOH solution again for 5 s, subjected to alkali treatment, and immersed in DI water at 90 °C for at least 10 min. Finally, it was dried in an oven at 175 °C to obtain a superhydrophilic mesh. This superhydrophilic mesh was coated with a self-assembled monolayer (SAM) using n-hexane and heptadecafluoro-1,1,2,2-tetraphydrodecyl trichlorosilane (C_10_H_4_C_l3_F_17_Si, HDFS, Gelest), and then dried in an oven at 175 °C to obtain a superhydrophobic mesh. The details of this surface modification process have been described in previous studies^29^.

**Table 1:** Specification of the base mesh used in this study.

Mesh	Wire diameter (mm)	Opening (mm)	Porosity (%)	Shade coefficient (SC)
#40	0.254	0.381	36	0.64

### Structure modification

**Figure 1:**
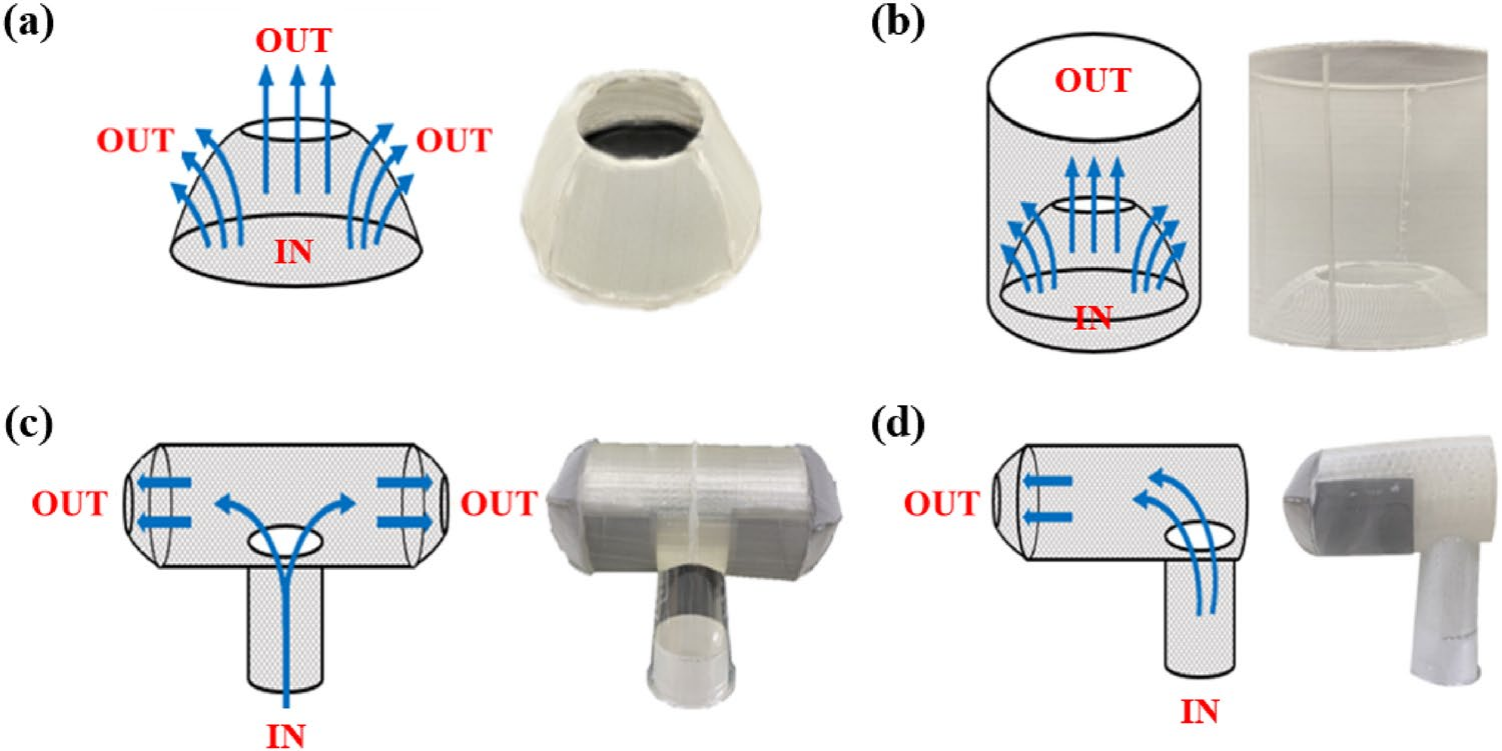
Collector shape modifications to improve fog harvesting efficiency. (**a**) Concave collector, (**b**) cylindrical mesh installed around the concave collector, (**c**) bidirectional collector, and (**d**) unidirectional collector. In the schematic diagram, gray parts represent meshes, and white parts represent spaces where fog passes through.

Fog harvesting efficiency can be calculated by considering the processes of capturing and draining, and is expressed as follows^7^:

1 $$\eta = {\eta }_{a} \cdot {\eta }_{c} \bullet {\eta }_{d},$$

where *η*_*a*_, *η*_*c*_, and *η*_*d*_ are the aerodynamic efficiency, capture efficiency, and draining efficiency, respectively. *η*_*a*_ represents the portion of droplets in undisturbed fog that can collide with the mesh. The theoretical equation for *η*_*a*_ is expressed as follows^7^:

2 $${\eta }_{a}= \frac{SC}{1+ \sqrt{{C}_{0}/{C}_{D}}},$$

where *SC* is the portion of the projection area covered by mesh fibers and is calculated as the clogging rate minus the porosity. *C*_*D*_ is the drag coefficient of the non-permeable mesh structure and is independent of *SC*. Equation (2) indicates that *η*_*a*_ is strongly affected by the collector shape and that a higher *C*_*D*_ yields a higher *η*_*a*_. *C*_*0*_ is the pressure loss coefficient of the mesh, which is affected by *SC*. For a wire mesh, the correlation for *C*_*0*_ proposed by Rivera is expressed as follows^7^:

3 $${C}_{0}=1.3SC+ {\left(\frac{SC}{1-SC}\right)}^{2}$$

where *C*_*0*_ of the meshes was 3.99. This *η*_*a*_ was not affected by the surface modification because the overall structure and *SC* did not change after the modification. The change in mesh wettability led to improvements in *η*_*c*_ and *η*_*d*_. It is known that the curved C-section has *C*_*D*_ of 2.3, but the *C*_*D*_ of rectangular plates with aspect ratio of 20 is 1.5^7^. Therefore, the curved mesh can improve *η*_*a*_ the resulting in overall harvesting efficiency. Therefore, in order to increase *C*_*D*_, a concave collector with a higher *C*_*D*_ than a flat structure was designed, as shown in Fig. 1a^7^. Curved meshes induce the deflection of uncaptured droplets and vortex flows. To improve harvesting performance further, a cylindrical mesh was installed around the concave collector, as shown in Fig. 1b.

**Figure 2:**
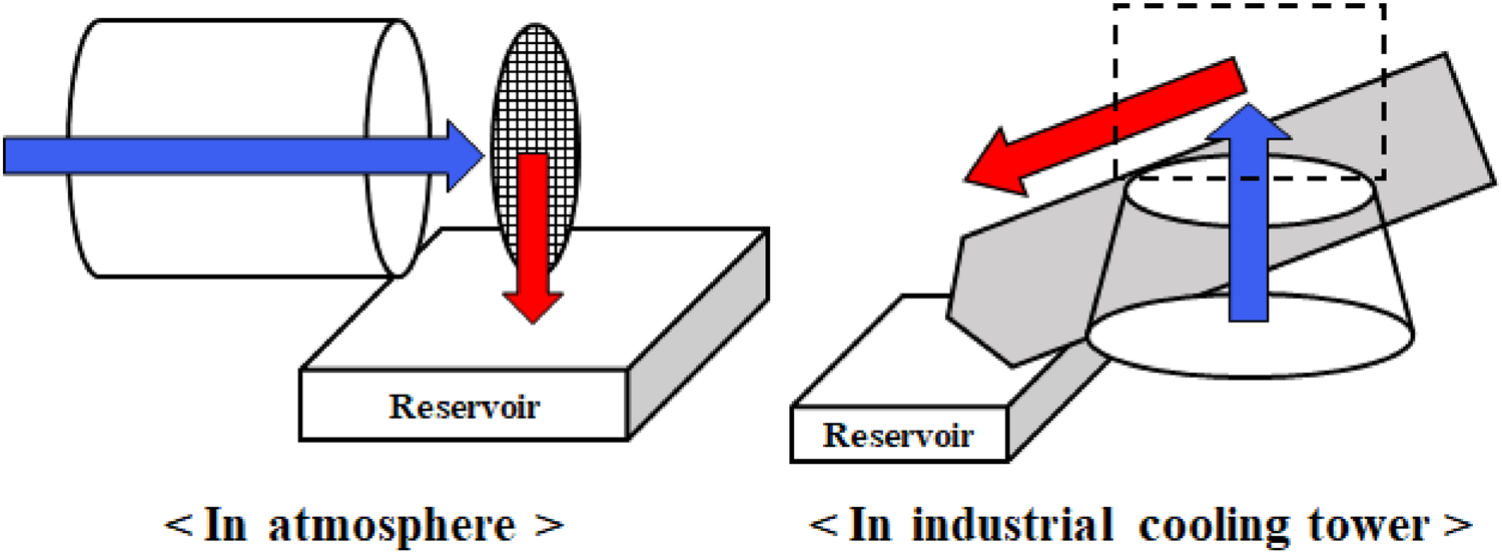
Schematic diagrams of water harvesting in the atmosphere (left) and in an industrial cooling tower (right).

Figure 2 presents a schematic diagram of water harvesting in the atmosphere and industrial cooling towers. In contrast to water harvesting in an atmosphere in which fog-laden flows are formed in a horizontal direction and collectors are placed vertically, the fog-laden flows in industrial cooling towers are typically formed vertically, and collectors are installed horizontally or inclined. This difference is one of the biggest causes of performance degradation in industrial cooling towers because droplets that fall back into towers under the influence of gravity are lost. Most droplets can be drained directly into a reservoir if they are captured in a fog capture net during atmospheric fog harvesting. However, based on the fundamental characteristics of industrial cooling towers, even if droplets are captured in a fog capture net, fallback loss may occur before droplets reach the drainage channel. Therefore, we propose a strategy for changing the discharged flow direction by forming an additional flow path similar to that of water harvesting in nature, as shown in Fig. 1c,d.

In order to change the flow direction, the additional flow paths were generated by connecting T-shaped and 90° bended channels to the outlet of cooling tower for bidirectional and unidirectional collectors, respectively. The channels were made was made of poly lactic acid (PLA) using a 3D printer. At the end of newly added horizontal channels, the concave collector, which was same with that in Fig. 1a, was attached to collect water.

### Measurement of industrial fog harvesting performance

**Figure 3:**
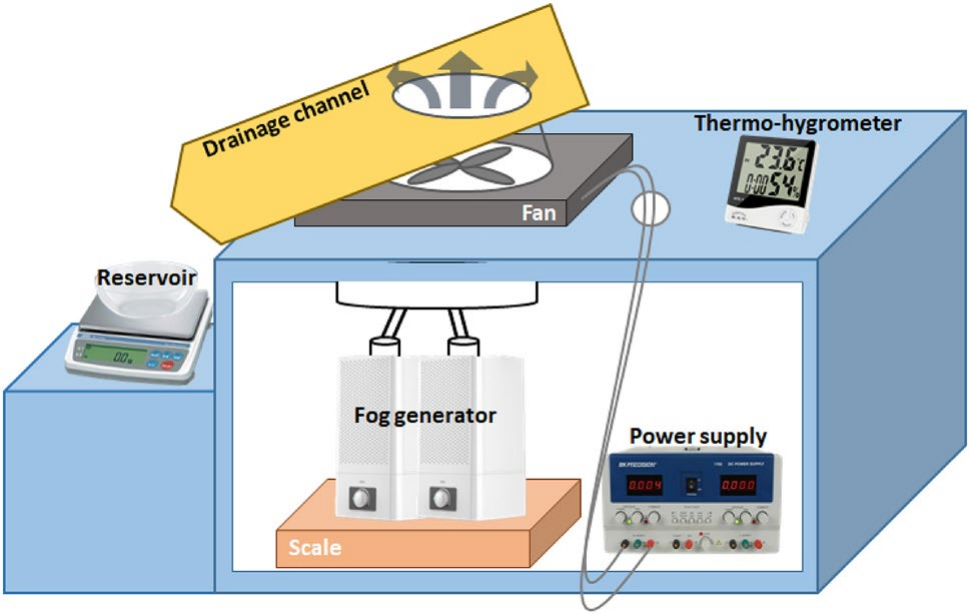
Schematic diagram of a scaled-down water harvesting experimental setup simulating fog-laden flows around industrial cooling towers.

Figure 3 presents a schematic diagram of a scaled-down water harvesting device that simulates the fog-laden flows around industrial cooling towers. A humidifier is used to generate supersaturated environmental conditions and the velocity of the discharged flow is controlled by adjusting the fan speed. The droplets deposited on the capture screen are stored in the final capture container, and the amount of water harvested is measured using a scale. The amount of fog generated is measured based on the weight reduction of the humidifier and the harvesting efficiency is calculated as the weight ratio of the collected water over the generated fog. The overall water collection efficiency is calculated as follows:

3 $$\upeta = \frac{{W}_{coll}}{{W}_{gen}}$$

The weight was measured every 30 min for 5 h. Without any collectors, the experiment was conducted to confirm the effect of the water collection by conduits instead of collector. Even though some water droplets attached conduits, there was no collection in the final reservoir where *W*_*coll*_ was measured. Therefore, the calculated *η* successfully represented the sole effect of designed collector. The uncertainty of the efficiency was only related to the measurement accuracy of weights. Two different balances were used for measuring the generated and collected water. SB60K11 (AND Company, limited, Korea) balance for generating water amount and FX-300i balance (AND Company, limited, Korea) for collecting water amount had an accuracy of 0.0003 and 0.001 g, respectively. The uncertainty was calculated from

4 $$\frac{\Delta\upeta }{\upeta }= \sqrt{{\left(\frac{\Delta {W}_{gen}}{{W}_{gen}}\right)}^{2}+{\left(\frac{\Delta {W}_{coll}}{{W}_{coll}}\right)}^{2}}$$

where Δ*W*_*gen*_ and Δ*W*_*coll*_ referred to the accuracy of used balances. The maximum uncertainty of the calculated *η* was 0.023%. Details of the calculation are given in the Table S1.

## Results and discussion

**Figure 4:**
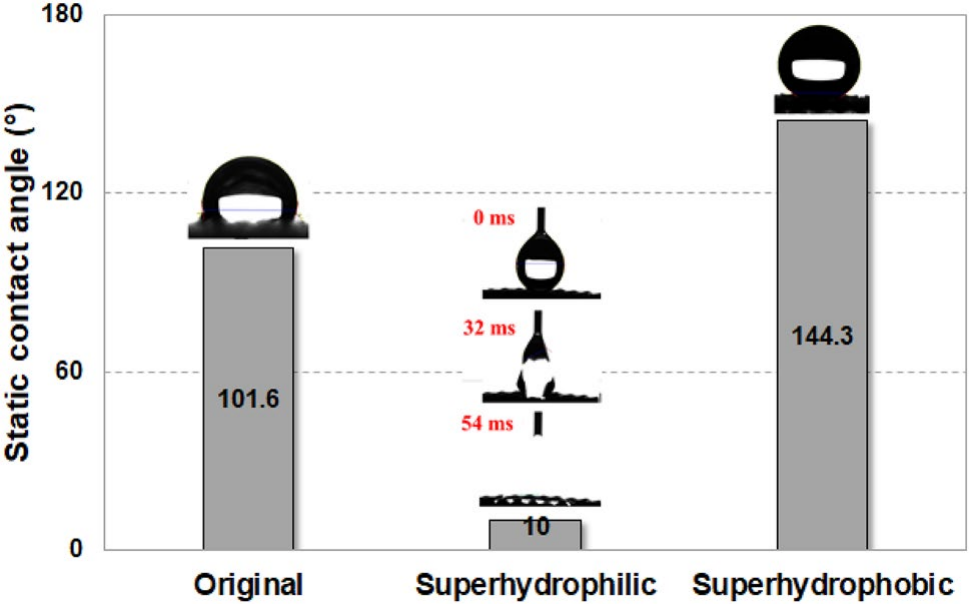
Comparison of contact angles for wettability-modified meshes.

The static contact angles of the original, superhydrophilic and superhydrophobic meshes are compared in Fig. 4. The contact angle of the Al mesh is 101.6°, which is similar to the contact angle of a typical smooth Al surface with weak hydrophilicity. As shown in Fig. 4, droplets on superhydrophilic mesh completely spread in a short time because of the increase in surface roughness after forming micro/nanostructures inducing the Wenzel state. The droplets on superhydrophobic mesh maintained spherical shapes, and the superhydrophobic mesh has a contact angle of 144.3° because droplets were unable to penetrate the air pocket due to HDFS SAM coating, inducing the Cassie state. In general, if the static contact angle is more than 150°, it is expressed as superhydrophobic^31^ In the case of superhydrophobic, it was measured to be less than 150° due to the mesh structures. Even though the static contact angle values were lower than 150°, the surface wettability of the meshes after SAM coating exhibited typical characteristics of superhydrophobic surfaces and could be classified as superhydrophobic^29^. Based on these contact angles, one can see that the surface wettability of the Al mesh was effectively modified. The characteristics of the wettability-modified meshes were described in our previous study^29^.

**Figure 5:**
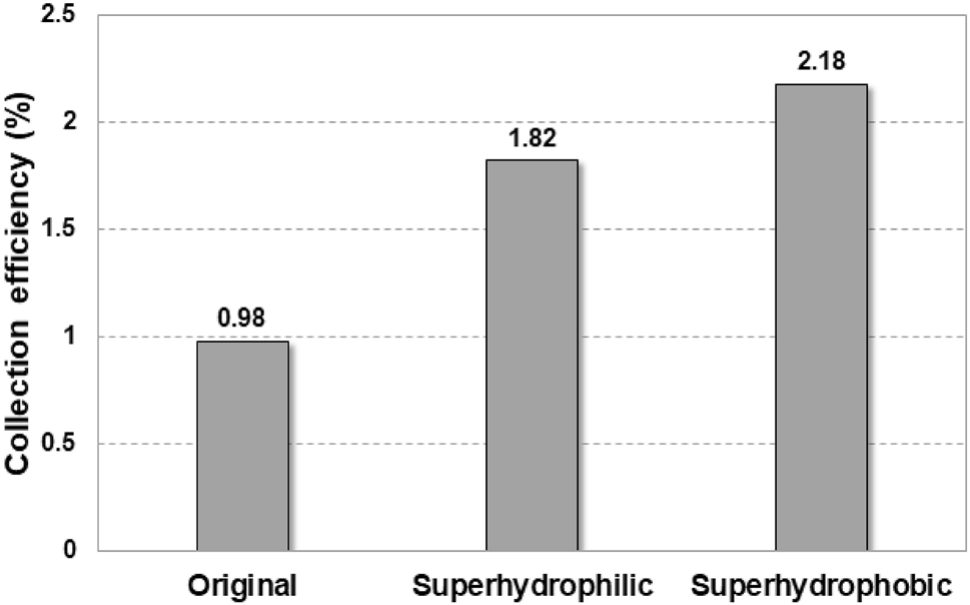
Effects of mesh wettability modification on the water harvesting efficiency of a flat oval collector at an inclined angle of 30°.

Our previous study demonstrate that the performance of industrial fog harvesting is strongly related to the inclination angle of the mesh screen because undesirable and premature dripping can occur, and it is maximized when the angle to the horizon is 30° with a flat square mesh^29^. The harvesting efficiencies of flat oval meshes with various levels of wettability at an inclined angle of 30° are compared in Fig. 5. The superhydrophobic mesh has the highest efficiency of 2.18% and the efficiencies of the superhydrophilic and original meshes are 1.82% and 0.98%, respectively. When water droplets are collected in the original mesh, pinning phenomena occurs, and the water droplets maintain a hemispherical shape. This causes not only the time increase of droplets traveling from the mesh to the reservoir but also the disturbance to the fog-laden flow. In contrast, droplets on the superhydrophilic mesh completely wet and clog pores. The clogged water attracts more droplets, and it grows into large droplets faster than the original mesh. This results in the faster growth to the critical size for drainage. Unlike the original and superhydrophilic meshes, clogging rarely appears in case of hydrophobic mesh, and rarely occurring clogged droplets have a small adhesion force due to the spherical suspension on mesh. This provides a quick drainage of the collected droplets. More details about droplet behaviors on meshes can be found in our previous study^29^.

**Figure 6:**
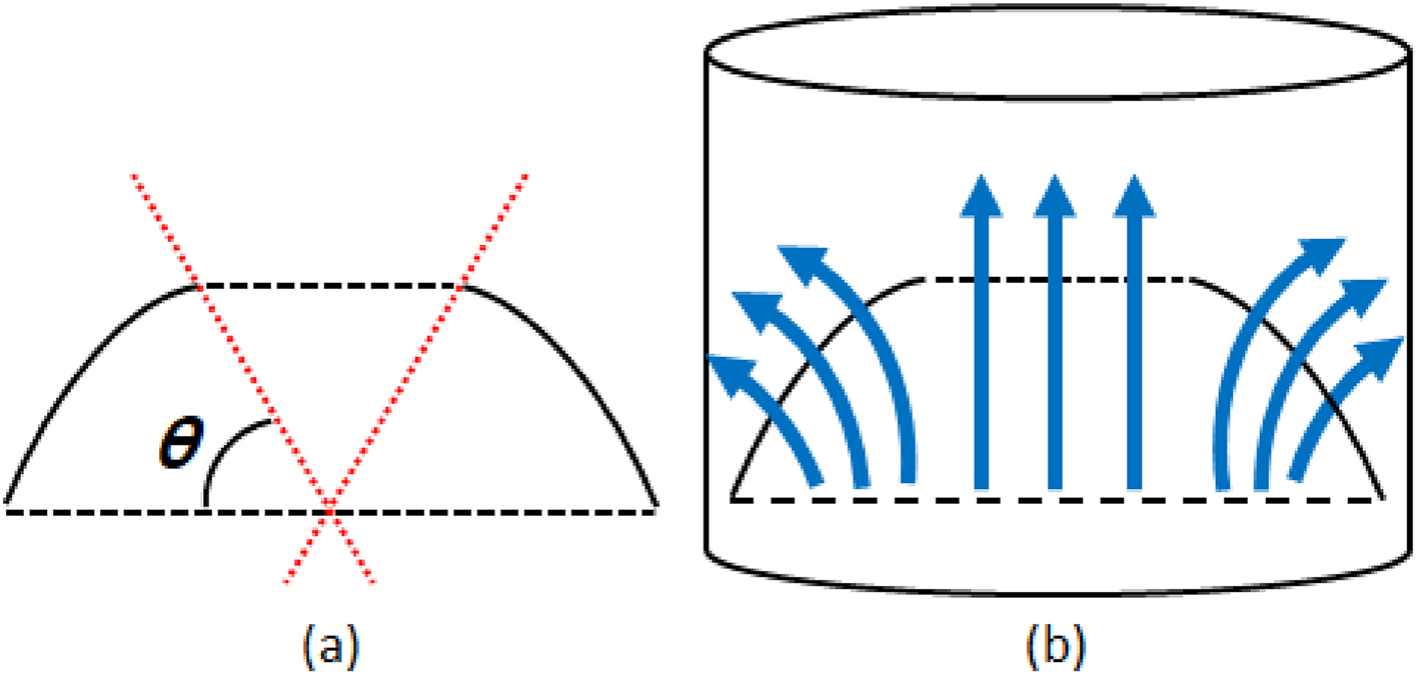
Detailed description of concave collector.

**Figure 7:**
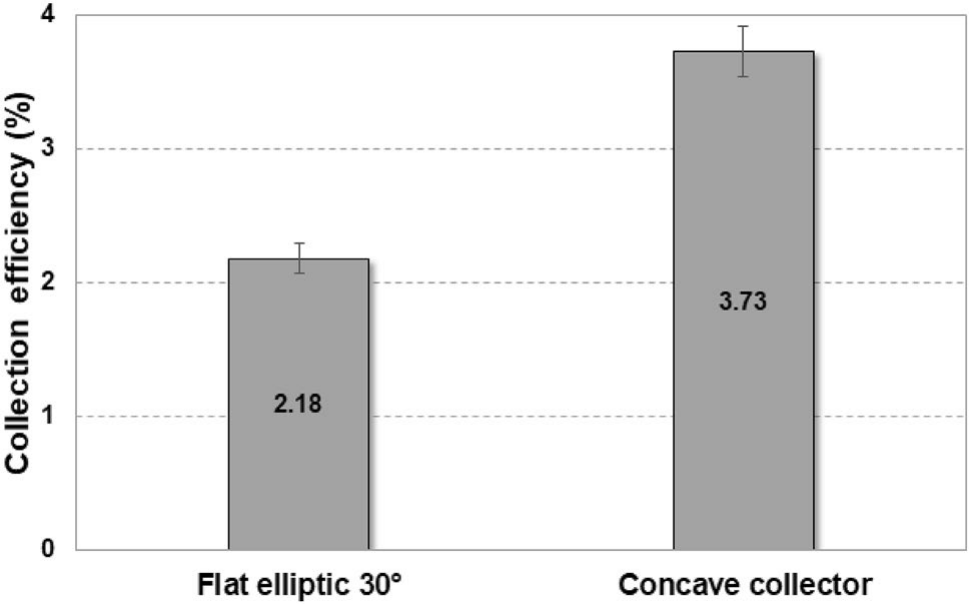
Comparison of the water harvesting efficiencies of a flat elliptic screen with a 30° inclined angle and a concave collector. Both structures contain superhydrophobic meshes.

Figure 6 shows a schematic diagram representing the detailed design of the concave collector. According to previous results about the inclined angle effect on harvesting efficiencies with plat meshes, the most effective angle to the horizontal line was 30°^29^. Therefore, in this study, we designed a concave collector with superhydrophobic meshes having an empty area (black dotted line) in which the inclination of the tangent is less than 30° (*θ* = 60°), as shown in Fig. 6a. The installation of a fog collector causes the resistance increase which requires more power for fog flows. The empty space part help to reduce the resistance that can reduce the additional consumption power of fan due to the water harvesting. Additionally, to increase the effective area, the meshes were placed on an optical shell (black line) instead of a circle. To investigate the effects of *C*_*D*_ on fog harvesting performance, the water harvesting efficiencies of the flat elliptic and proposed concave collectors with the same projected areas were compared, as shown in Fig. 7. The concave collector has an efficiency of 3.73%, which is 1.71 times greater than that of the flat elliptic mesh. As expected, based on the theoretical model in Eq. (2), a concave collector with a high *C*_*D*_ improves the overall fog harvesting efficiency by increasing *η*_*a*_.

To investigate the effects of a curved surface on fog-laden flows, velocity field information was measured using particle image velocimetry (PIV). PIV provides velocity field information over large planar regions and is suitable for investigating flow structures^32–34^. Droplets in fog-laden flows were used as tracers and droplet images were captured using a high-speed camera (Fastcam Mini Ux50, Photron, USA). An 8 W diode-pumped solid-state continuous laser (Changchun New Industries Optoelectronics Technology Co., China) with a wavelength of 532 nm was used to generate a light sheet. The final velocity vectors were obtained by processing images using open-source software for performing PIV analysis in MATLAB^35^.

**Figure 8:**
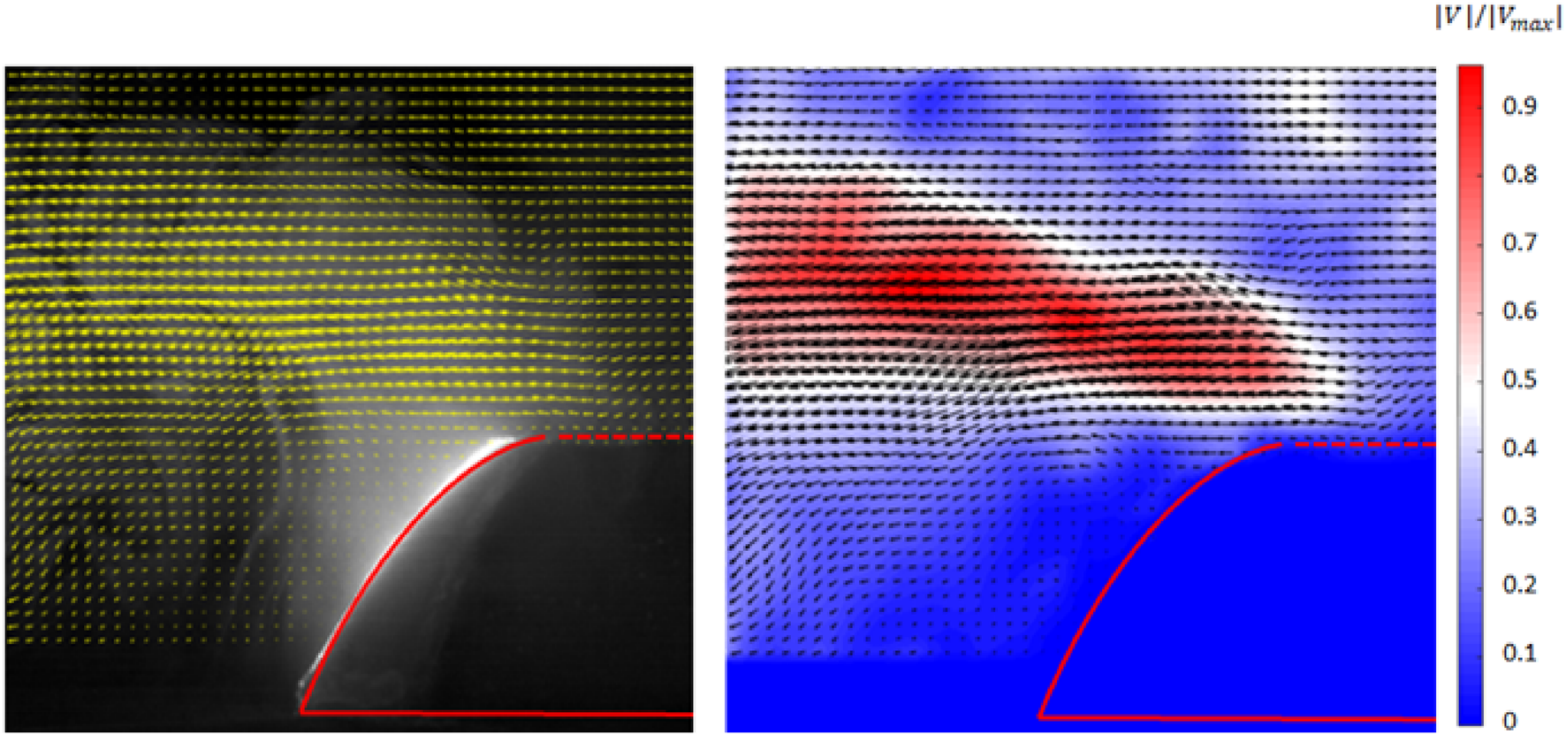
Velocity vectors (left) and velocity magnitude contours (right) of fog-laden flows with a concave collector.

**Figure 9:**
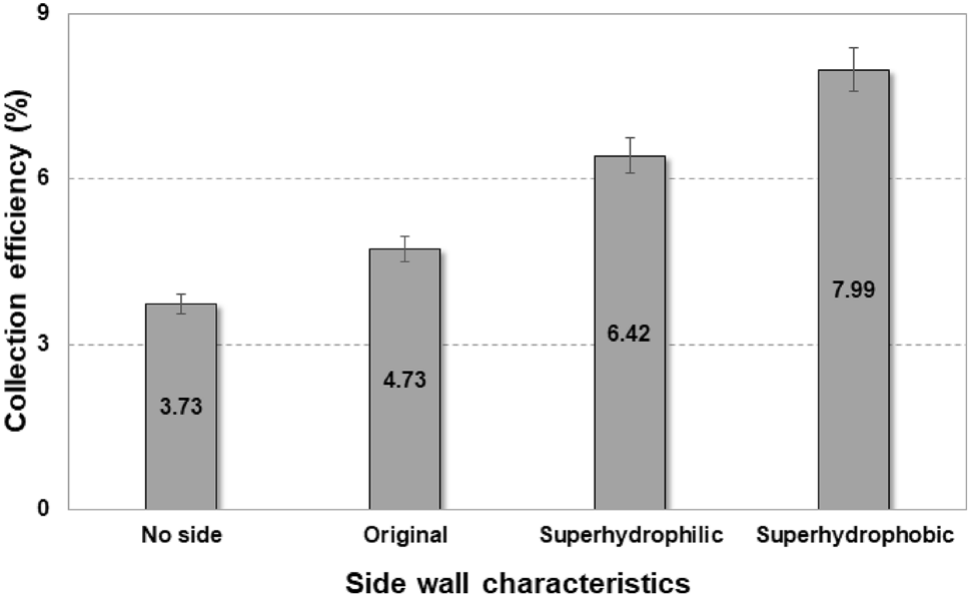
Comparison of water harvesting efficiencies after installing side walls on a concave collector.

The velocity field presented in Fig. 8 confirms that flow deflection occurs as a result of the concave collector and a large number of uncollected droplets flow sideways. The formation of a small-scale vortex flow can also be observed in the PIV results. It was expected that fog harvesting efficiency could be significantly improved by collecting these deflected droplets. To collect uncollected droplets in deflected fog-laden flows, a cylindrical mesh was incorporated, as shown in Fig. 6b. The wettability of this side mesh was also modified and its performance is presented in Fig. 9. When the droplet carry-over loss in superhydrophobic mesh can occur when the fog-laden flow velocity is high enough. As shown in Fig. 8, the high velocity region was started from the upper edge of the concave collector, and the high velocity propagated laterally upward. This represented that the carry-over loss could be significant in side wall with superhydrophobic mesh. Therefore, various meshes having different wettability were applied to the side wall. Figure 9 indicates that the additional side mesh on the concave collector significantly improves harvesting performance by a factor of at least 1.27, even without any wettability modification. Modification of the side mesh wettability was also effective, and the fog harvesting efficiencies for superhydrophilic and superhydrophobic meshes were 6.42% and 7.99%, respectively. This shows that the carry-over loss was not significant under this experimental conditions. Superhydrophobic modifications for both the concave collector and side mesh provide the maximum fog harvesting efficiency. In this study, we considered not only a mesh, but also an Al flat sheet as a side screen, but the sheet exhibited much lower efficiency.

**Figure 10:**
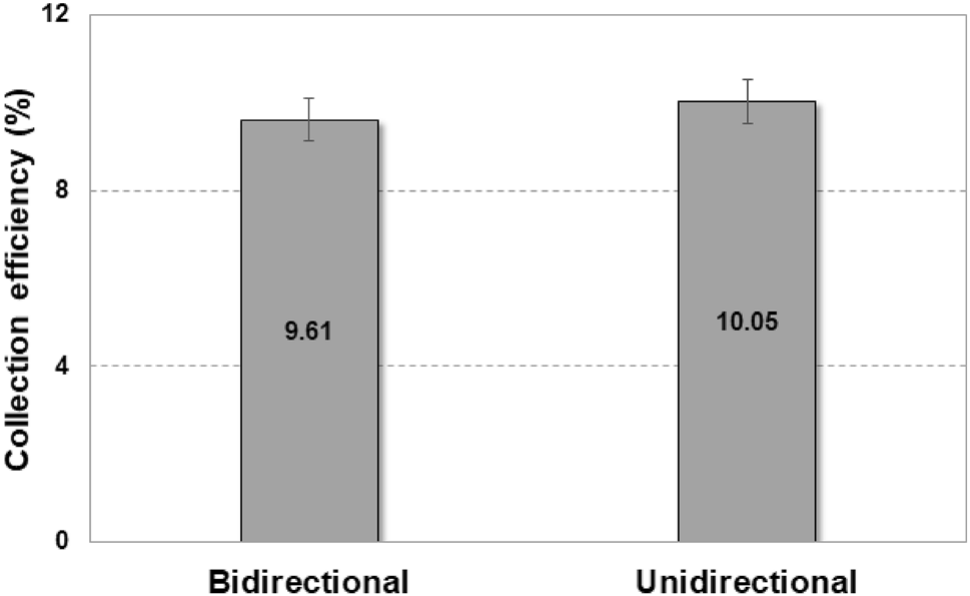
Comparison of water harvesting efficiencies in an industrial cooling tower with an additional flow path for changing the discharged flow direction.

Although the efficiency was significantly improved, it was still low compared with the harvesting performance in nature, where the maximum fog harvesting efficiency was measured to be 12.02% in our previous study^29^. As mentioned previously, this difference can be attributed to the directions of the fog-laden flows. Figure 10 presents the harvesting efficiency with the incorporation of an additional flow path for changing the flow direction. A concave collector was attached at the exit of the additional flow path. The harvesting efficiencies are 9.61% when the discharge flow path is bidirectional and 10.05% when it travels only in one direction. This change in the discharge direction results in an increase in harvesting performance and there is little difference compared to the maximum efficiency of atmospheric fog harvesting. This indicates that if the direction of discharged flows from cooling towers is changed from vertical to horizontal, then the fallback loss generated during drainage can be reduced and water harvesting efficiency can be improved. Additionally, when the discharge direction is unidirectional, rather than bidirectional, the amount of loss along the flow path wall can be reduced, resulting in an improved water harvesting performance.

## Conclusion

In this study, a simple and economical method for collecting freshwater was designed to solve water shortage problems. Fog collection in nature has been studied extensively in previous studies, but fog collection in industrial cooling towers has received relatively little attention. Additionally, there have been no studies on the effects of collector structures on the performance of industrial fog harvesting. To improve fog collection performance, the mesh surface characteristics and fog collector structure were modified, and the harvesting performance was experimentally evaluated. The following conclusion can be drawn.

- A concave shape increases the drag coefficient resulting in the improved fog harvesting performance compared to a flat mesh.
- Fog harvesting performance can be drastically improved by collecting droplets in deflected fog-laden flows around a concave shape by incorporating a side wall.
- The change of the discharge direction to horizontal can provide higher harvesting performances by reducing fallback losses during the drainage of droplets.
- If it is impossible to change the flow path, a concave collector with a superhydrophobic side wall is an optimal design for the fog harvesting from industrial cooling towers.
- Although the effectiveness of the proposed method was successfully validated in well-controlled laboratory-scale tests, unexpected problems with a scale-up design can occur under actual environmental conditions. Therefore, in the next future study, we will try to apply the proposed method to actual cooling towers and optimize the shape of the collector to be simpler and compact for practical applications.

The shapes proposed in this study can contribute significantly to the improvement of fog harvesting performance in the future and are expected to contribute to resolving social conflicts and shortages of freshwater.

## Supplementary Information

Supplementary Table S1.
