# Supplementary material for: Improvement of water harvesting performance through collector modification in industrial cooling tower
Source: Sci Rep. 2022 Mar 18;12:4658. doi: 10.1038/s41598-022-08701-3 (PMC8933487; doi:10.1038/s41598-022-08701-3)
Supplement: Supplementary file 1 — Supplementary Table S1. [file 41598_2022_8701_MOESM1_ESM.docx]

Table S1. Maximum calculated uncertainty for the harvesting efficiency

| Collector | Flat oval collector  (inclined angle : 30°) | | | Concave  collector | | | Side walls  on a concave collector | | | Discharged  flow direction | | |
| --- | --- | --- | --- | --- | --- | --- | --- | --- | --- | --- | --- | --- |
|  | **OM** | **SHPM** | **SHBM** | | **SHBM** | **OM** | | **SHPM** | **SHBM** | | **Bi-** | **Uni-** |
| W_gen_ [g/hr] | 445.56 | 491.11 | 1840 | | 396.17 | 462.5 | | 435 | 431.94 | | 458.07 | 416.20 |
| W_coll_ [g/hr] | 4.33 | 8.78 | 40.1 | | 14.99 | 21.53 | | 28.14 | 35.05 | | 41.81 | 35.84 |
| $\boldsymbol{\eta}$ [%] | 0.98 | 1.82 | 2.18 | | 3.73 | 4.73 | | 6.42 | 7.99 | | 9.61 | 10.05 |
| $\frac{\boldsymbol{\Delta}\boldsymbol{W}_{\boldsymbol{gen}}}{\boldsymbol{W}_{\boldsymbol{gen}}}$ | 5.E-13 | 4.E-13 | 3.E-14 | | 6.E-13 | 4.E-13 | | 5.E-13 | 5.E-13 | | 4.E-13 | 5.E-13 |
| $\frac{\boldsymbol{\Delta}\boldsymbol{W}_{\boldsymbol{coll}}}{\boldsymbol{W}_{\boldsymbol{coll}}}$ | 5.E-08 | 1.E-08 | 6.E-10 | | 4.E-09 | 2.E-09 | | 1.E-09 | 8.E-10 | | 6.E-10 | 8.E-10 |
| Uncertainty [%] | 0.0231 | 0.0114 | 0.0025 | | 0.0067 | 0.0046 | | 0.0036 | 0.0029 | | 0.0024 | 0.0028 |
| 1. OM : Original mesh 2. SHPM : Superhydrophilic mesh 3. SHBM : Superhydrophobic esh 4. Bi- : Bidirectional collector 5. Uni- : Unidirectional collector 6. W_gen_ : Generating amount 7. W_coll_ : Collecting amount 8. $\boldsymbol{\eta}$ : Collection efficiency | | | | |  |  | |  |  | |  |  |
